# Motion processing: the most sensitive detectors differ in temporally localized and extended noise

**DOI:** 10.3389/fpsyg.2014.00426

**Published:** 2014-05-15

**Authors:** Rémy Allard, Jocelyn Faubert

**Affiliations:** ^1^INSERM, U968Paris, France; ^2^Institut de la Vision, UMR_S 968, Sorbonne Universités – Université Pierre-et-Marie-CurieParis, France; ^3^CNRS, UMR_7210Paris, France; ^4^Visual Psychophysics and Perception Laboratory, Université de Montréal, Montréal, QCCanada; ^5^NSERC-Essilor Industrial Research Chair, Montreal, QCCanada

**Keywords:** local noise, extended noise, motion, detection, discrimination

## Abstract

Contrast thresholds for discriminating orientation and direction of a drifting, oriented grating are usually similar to contrast detection thresholds, which suggest that the most sensitive detectors are labeled for both orientation and direction (Watson and Robson, 1981). This was found to be true in noiseless condition, but Arena et al. (2013) recently found that this was not true in localized noise (i.e., noise having the same spatiotemporal window as the target) as thresholds for discriminating direction were higher than for discriminating orientation. They suggested that this could be explained by the fact that there are more neurons selective to orientation than direction. Another possible interpretation is that, unlike contrast thresholds in absence of noise, the most sensitive detectors in localized noise were labeled for orientation, but not for direction. This hypothesis is supported by recent findings showing different processes operating in localized and extended noise (i.e., full-screen, continuously displayed noise, Allard and Cavanagh, 2011). In the current study, we evaluated contrast thresholds for orientation and direction discrimination tasks in noiseless conditions, and in noise that was either spatially localized or extended, and temporally localized or extended. We found similar orientation and direction thresholds in absence of noise and in temporally extended noise, but greater direction thresholds in temporally localized noise. This suggests that in noiseless and temporally extended noise the most sensitive detectors were labeled for both orientation and direction (e.g., direction-selective complex cells), whereas in temporally localized noise the most sensitive detectors were labeled for orientation but not direction (e.g., simple cells). We conclude that to avoid violating the noise-invariant processing assumption, external noise paradigms investigating motion processing should use noise that is temporally extended, not localized.

## INTRODUCTION

Arena et al. (2013) used an external noise paradigm to investigate age-related sensitivity losses to motion processing by measuring contrast thresholds for discriminating either the orientation or the direction of drifting gratings. When the dominating noise source was internal because external noise had a negligible impact (i.e., in low noise), they observed an age-related sensitivity loss for both tasks, which could be due, according to the linear amplifier model (Pelli, 1981; Pelli and Farell, 1999), to an increase in internal equivalent noise (e.g., more internal noise with aging) or a decrease in calculation efficiency (i.e., greater signal-to-noise ratios required to perform the tasks with aging). Conversely, when internal noise had a negligible impact because the dominating noise source was external (i.e., in high noise), the two age groups had similar contrast thresholds and thereby necessitated similar signal-to-noise ratios to perform the tasks (i.e., they had similar calculation efficiencies). By implicitly assuming that the calculation efficiency in low noise was the same as the measured calculation efficiency in high noise (i.e., the noise-invariant processing assumption underlying external noise paradigms, Allard and Cavanagh, 2011), they concluded that the age-related sensitivity losses in low noise were due to an increase in internal equivalent noise, not a decrease in calculation efficiency.

However, their data actually suggest that different processes operated in low and high noise, which would invalidate the assumption that the calculation efficiencies in low noise were the same as the measured calculation efficiencies in high noise. In low noise, similar contrast thresholds were observed for discriminating orientation and direction, which suggests that both measured the sensitivity of the same processing system having its most sensitive detectors labeled for both orientation and direction (Watson and Robson, 1981). In high noise, however, contrast thresholds for orientation discrimination were lower than for direction discrimination suggesting that the most sensitive detectors were labeled for orientation, but not direction. Consequently, in low noise the most sensitive detectors would be labeled for both orientation and direction (e.g., direction-selective complex cells), but in high noise they would only be labeled for orientation, not for direction (e.g., simple cells). If contrast thresholds depended on the sensitivity of different detectors in low and high noise, then the assumption that the calculation efficiency in low noise was the same as the measured calculation efficiency in high noise would be compromised and without knowing the calculation efficiency in low noise it is not possible to determine if the age-related sensitivity loss in low noise was due to an increase in internal equivalent noise or a decrease in calculation efficiency.

As in many studies, Arena et al. (2013) used spatiotemporally localized noise appearing only at the spatiotemporal target location (personal communication), which could explain that the most sensitive detectors were not the same in low and high noise. Indeed, given that internal noise (which dominates in low noise) is spatiotemporally extended (e.g., it does not turn on and off with the stimulus and it is not located only at the stimulus location), the dominating noise source in low and high localized noise have different spatiotemporal windows: extended in low noise and localized in high noise. If the most sensitive detectors differ depending on whether the dominating noise source is localized or extended, this would cause the most sensitive detectors to differ in low and high localized noise, which would compromise the assumption that the calculation efficiency in low noise (i.e., in extended internal noise) is the same as the measured calculation efficiency in high localized noise. For instance, noise that is temporally localized to the target (i.e., turn on and off with the target) introduces strong onset and offset transients, which could result in a greater masking effect on direction selective detectors making detectors labeled for orientation more sensitive than detectors labeled for both orientation and direction.

The objective of the present study was to determine if the processes (e.g., most sensitive detectors) involved in discriminating the orientation and the direction of drifting gratings in localized and extended noise differ from the processes operating in absence of noise. More specifically, the goal of the current study was to determine whether the calculation efficiencies in absence of noise (i.e., in extended internal noise) differ for orientation and direction discrimination (as observed by Arena et al. in high localized noise) or not (as suggested by the similar contrast thresholds in low noise). To investigate this, we conducted an experiment similar to Arena et al.’s (2013) in which contrast thresholds were measured for discriminating orientation and direction in absence of noise (i.e., in extended internal noise) and in high noise having different spatiotemporal windows: spatially localized or extended and temporally localized or extended. Given that contrast threshold depends on both the dominating noise source and the calculation efficiency (i.e., signal-to-noise ratio required to perform the task) and that the level of the dominating noise source is known in high noise, calculation efficiency in high noise can be directly measured by measuring contrast threshold in high noise. If the calculation efficiencies in absence of noise (which cannot be directly measured because the internal noise level is unknown) are the same for orientation and direction discrimination, but differ in high localized noise (as measured by Arena et al., 2013) due to the noise being localized, then we would expect the calculation efficiencies in high extended noise to be similar for orientation and direction discrimination. This would show a violation of the noise-invariant processing assumption when using localized noise as the calculation efficiencies measured in localized noise would not reflect the calculation efficiencies in absence of noise. Conversely, if the calculation efficiency in absence of noise is greater for orientation discrimination (as measured in high localized noise by Arena et al., 2013), then the calculation efficiency for orientation discrimination should also be greater in high extended noise. For instance, Arena et al. (2013) hypothesized that the calculation efficiency difference between the two tasks in high localized noise could be due to more neurons responding to orientation than to direction or to the fact that discriminating direction requires more spatiotemporal integration than discriminating orientation. In either case, a similar calculation efficiency (i.e., contrast threshold) difference would also be expected in extended noise.

The current study is not the first to question the use of localized noise within external noise paradigms. Allard and Cavanagh (2011) found that crowding impaired contrast detection in the near periphery in localized noise, but not in absence of noise or in extended noise. We found that aging can impair contrast thresholds in localized noise, but not in extended noise (Allard et al., 2013). Furthermore, we recently argued that using spatiotemporally localized noise that is also localized as a function of orientation and frequency (i.e., contains only the orientation and frequency of the stimulus) makes a contrast detection task switch to a contrast discrimination task (Allard and Faubert, 2013). All these studies focused on the contrast detection of a static target. However, because localized noise introduces strong transients, using localized noise could be even more critical for motion processing. The current study addresses this question using a different paradigm that more directly identifies the underlying process (compared to crowding or aging) by determining if the most sensitive detectors are direction selective or not.

## MATERIALS AND METHODS

### OBSERVERS

Three naïve observers, who were financially compensated and provided informed consent, and one of the authors, participated in this study. They had normal or corrected-to-normal vision.

### APPARATUS

The stimuli were presented on a 19-inch CRT monitor with a refresh rate of 120 Hz. The Noisy-Bit method (Allard and Faubert, 2008) implemented independently to each gun made the 8-bit display perceptually equivalent to an analog display having a continuous luminance resolution. The monitor was the only source of light in the room. A Minolta CS100 photometer interfaced with a homemade program calibrated the output intensity of each gun. At the viewing distance of 114 cm, the width and height of each pixel were 1/64° of visual angle.

### STIMULI AND PROCEDURE

The signal was a 0.5 cpd sine wave grating drifting at a frequency of 1.875 Hz. Observers were asked to report either the orientation (tilted either -45 or 45° from vertical) or the drifting direction. When the task was to report the orientation, both the orientation (-45 or 45°) and direction were randomized. When the task was to report the drifting direction, the orientation was fixed for a given block of trials and the drifting direction was randomized. The initial phase of the grating was randomized on each trial. The signal was presented for 267 ms. The spatial window was a circular aperture of 4° plus a half-cosine edge of 0.5°. The contrast was controlled by a 3-down-1-up staircase procedure (Levitt, 1971) with step size of 0.1 log, which was interrupted after 100 trials. The contrast threshold for a given staircase was estimated as the geometric mean of the inversions.

There were five different noise conditions: no noise and four noise conditions resulting from the combinations of two spatial and two temporal windows. The spatial window was either localized or extended, i.e., the same as the signal window or full-screen, respectively. The temporal window was also either localized or extended, i.e., turn on and off with the signal or continuously present (including between trials), respectively. The noise was binary with element size of 4 × 4 pixels (i.e., 0.0625 × 0.0625°) and resampled every other frame (i.e., dynamic at 60 Hz). Thus, the fact that the noise was not correlated over space (across noise elements) and time (across frames) implies that it was both temporally and spatially white, that is, it had the same spectral energy at all frequencies (within the limit of the spatial and temporal resolution of the noise). The noise was superimposed to the signal (both summed) and to avoid luminance motion drifting cues within noise elements, there was no spatial or temporal luminance variation within each noise element.

For each noise condition, contrast thresholds were estimated for direction and orientation discrimination. To perform the same number of measurements for orientation and direction discrimination, a given noise block contained four staircases: direction discrimination for the two orientations (-45 and 45°) and two identical orientation discriminations. The four staircases were blocked and tested in a random order. Each of the five noise blocks was tested twice in a pseudo-random order resulting in 10 noise blocks (two blocks per noise condition) each composed of four staircases (two for direction discrimination and two for orientation discrimination) performed in a random order (not interlaced). As a result, for each noise condition, the two contrast threshold estimations were based on the geometric mean of the contrast thresholds estimations based on four staircases.

## RESULTS

**FIGURE 1:**
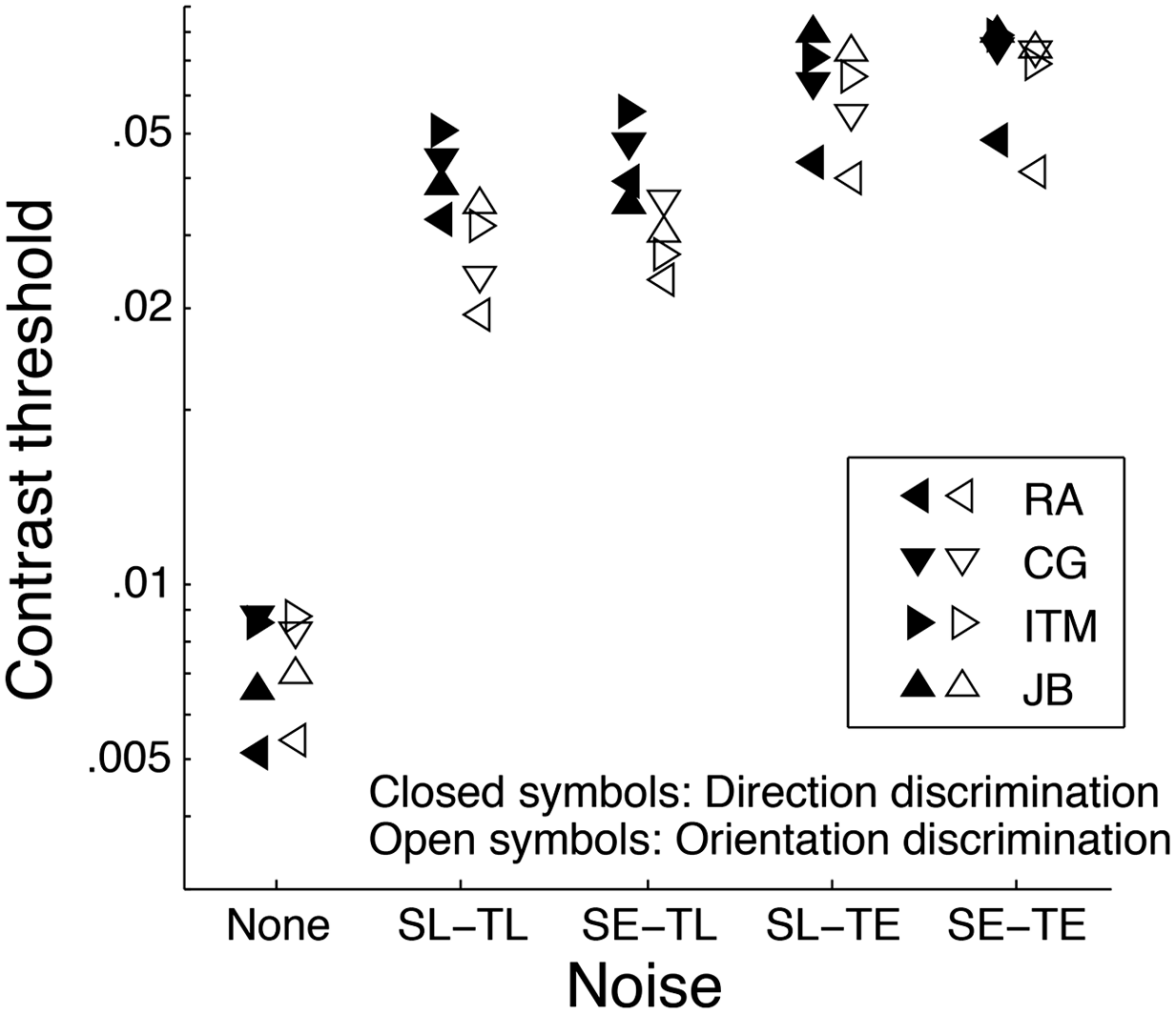
**Contrast thresholds obtained for four subjects (different symbols) in five different noise conditions for orientation (open symbols) and direction (closed symbols) discrimination.** In the four noise conditions, the noise was either spatially localized (SL) or extended (SE) and temporally localized (TL) or extended (TE). Each data point corresponds to the average of four staircases. For clarity, the standard error are not shown, but were all smaller than 0.06 log units (i.e., less than a factor of 1.15).

**Figure 1** shows contrast thresholds for orientation (open symbols) and direction (filled symbols) discrimination. Contrast thresholds in the four conditions with noise were substantially higher (by a factor of about 4) than the condition without noise. This confirms that these four conditions were performed in high noise, that is, the impact of internal noise was negligible (i.e., the dominating noise source was external) so that contrast thresholds therefore depended solely on calculation efficiency, not on internal equivalent noise. Contrast thresholds were roughly unaffected by the noise *spatial* window as similar contrast thresholds were observed in spatially localized and extended noise both when the temporal window was localized (SL-TL and SE-TL) and extended (SL-TE and SE-TE). This was statistically validated by a 2 × 2 × 2 ANOVA (task × spatial window × temporal window), which revealed no significant effect of spatial window [*F*(1,3) = 1.83, *p* = 0.27] and no task × spatial window interaction [*F*(1,3) = 0.019, *p* = 0.90]. On the other hand, contrast thresholds varied with the noise *temporal* window [*F*(1,3) = 57.9, *p* < 0.01] and varied differently for the two tasks (task × temporal window interaction, *F*(1,3) = 10.4, *p* < 0.05). Specifically, contrast thresholds were lower (i.e., higher calculation efficiency) in temporally localized noise (SL-TL and SE-TL) relative to temporally extended noise (SL-TE and SE-TE, respectively) by a factor of about 2 for orientation discrimination and 1.4 for direction discrimination.

**FIGURE 2:**
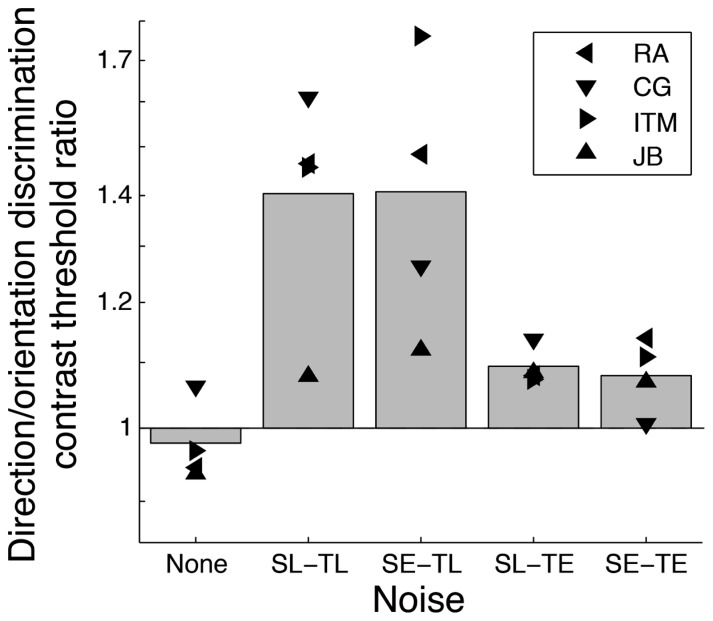
**Contrast threshold ratios for direction discrimination relative to orientation discrimination derived from **Figure 1** for four subjects (different symbols)**. A value of 1 represents the same threshold for both tasks. A value greater than 1 means that contrast thresholds were higher (or calculation efficiencies were lower) for discriminating direction compared to orientation.

**Figure 2** illustrates the contrast threshold ratios for direction relative to orientation discrimination represented in **Figure 1**. Similar contrast thresholds were observed for orientation and direction discrimination (i.e., ratios close to 1) in absence of noise and in temporally extended noise (SL-TE and SE-TE), but contrast thresholds were substantially better (by a factor of about 1.4 on average) for orientation than for direction discrimination in temporally localized noise (SL-TL and SE-TL, respectively).

## DISCUSSION

Calculation efficiency ratios (which can be directly inferred from contrast threshold ratios in high noise) of direction discrimination relative to orientation discrimination varied with the noise temporal window: a substantial difference was observed in temporally localized noise (threshold ratio of ∼1.4), but not in temporally extended noise (ratio close to 1). The purpose of external noise paradigms is generally to estimate the calculation efficiency in absence of noise by assuming that it is the same as the measured calculation efficiency in high noise. However, the fact that the calculation efficiency ratios varied with the noise temporal window implies that in at least one condition the measured calculation efficiency in high noise did not correspond to the calculation efficiency in absence of noise. Indeed, the calculation efficiency in absence of noise cannot both differ substantially for orientation and direction discrimination as measured in localized noise and be similar for orientation and direction discrimination as measured in extended noise. Thus, in at least one condition, the calculation efficiency measured in high noise did not correspond to the calculation efficiency in absence of noise, which violates the noise-invariant processing assumption and compromises the application of the external noise paradigm.

In absence of noise (i.e., in internal noise), no substantial contrast threshold difference was observed (ratios close to 1) as in temporally extended noise. Given that internal noise is expected to be temporally extended (it does not turn on and off with the stimulus) and that contrast thresholds were similar for orientation and direction discrimination as in extended noise, this suggests that the calculation efficiencies in absence of noise did not differ between tasks. As a result, there was no evidence of a violation of the noise-invariant processing assumption when using temporally extended noise so the calculation efficiency measured in temporally extended noise likely reflects the calculation efficiency in absence of noise. Contrariwise, the facts that internal noise is not temporally localized and that a different pattern of results was observed in temporally localized noise suggest that the calculation efficiencies measured in temporally localized noise were not both the same as the calculation efficiencies in absence of noise. This shows a violation of the noise-invariant processing assumption, as the measured calculation efficiency in localized noise cannot be assumed to be the same as the calculation efficiency in absence of noise.

The results of the current study suggest that when temporally localized noise dominated the most sensitive detectors were labeled for orientation only (e.g., simple cells), whereas when temporally extended noise dominated (which includes internal noise) the most sensitive detectors were labeled for both direction and orientation (e.g., direction-selective complex cells). Thus, which detectors were the most sensitive depended on the temporal window of the dominating noise source. This suggests that temporally localized noise impaired more the sensitivity of detectors labeled for orientation and direction (e.g., direction-selective complex cells, which would be the most sensitive in absence of noise) than the ones labeled for orientation only (e.g., simple cells). This greater masking for direction selective detectors can be explained by the sharp contrast transient onset and/or offset of the noise. Note that technically, there is more luminance transient between two different noise frames than between a mean gray frame and a noise frame. However, the temporal envelope of the localized noise contains a strong transient (turns on and off, i.e., noise contrast varies from 0 to high to 0) whereas the extended noise does not (it is continuously present, i.e., constant mean contrast). This corresponds to the subjective impression: temporally localized noise suddenly appears causing a sharp transition from a blank to a noisy display whereas temporally extended noise appears to be constantly displayed even if it is dynamic. Thus, the current results suggest that the sharp transients of the noise envelope (i.e., noise onset and offset) impair more the detectors labeled for both orientation and direction than the ones labeled for orientation only.

Given that transients caused by localized noise cause additional masking, one could expect thresholds to be lower (i.e., better) in extended noise than in localized noise, which is opposite to the current findings (**Figure 1**). Even though adaptation is known to reduce responsiveness of stimulated cells (Giaschi et al., 1993), it is unlikely that it affects contrast threshold in high noise because adaptation would affect the responses related to both the signal and noise leaving the signal-to-noise ratio intact. This would have no impact on contrast threshold given that contrast threshold in high noise is proportional to the noise contrast (Pelli, 1981). Conversely, there are at least two reasons why extended noise could have a greater masking effect than localized noise. First, the visual system has a limited temporal resolution and therefore integrates some noise outside the signal temporal window (i.e., just before the target onset and after the target offset). Second, localized noise has the advantage of reducing temporal uncertainty, which is obviously not the case for temporally extended noise. Thus, adding noise outside the temporal window of the signal (i.e., passing from localized to extended noise) can facilitate contrast threshold by removing noise onset and offset transient, but impair contrast threshold by introducing more noise and increasing temporal uncertainty. It is therefore not surprising that contrast thresholds in temporally extended noise are higher than in temporally localized noise even though there is no noise onset and offset transient in extended noise.

By compromising the estimation of the calculation efficiency in absence of noise, a violation of the noise-invariant processing assumption also compromises the estimate of the internal equivalent noise. Based on the linear amplifier model (Pelli, 1981; Pelli and Farell, 1999), contrast threshold in absence of noise depends on both internal equivalent noise and calculation efficiency. By knowing the contrast threshold in absence of noise and by assuming that the calculation efficiency in absence of noise is the same as the measured calculation efficiency in high noise, the internal equivalent noise can be calculated. If the calculation efficiency in absence of noise cannot be assumed to be the same as the measured calculation efficiency in high noise, then the internal equivalent noise cannot be calculated. For instance, Arena et al. (2013) observed that aging affected contrast thresholds in low, but not in high, localized noise. Given that contrast thresholds in high noise depend only on the calculation efficiency and not on the internal equivalent noise, they concluded that the calculation efficiency in low noise was not affected with aging and therefore attributed the age-related sensitivity losses in low noise to an increase in internal equivalent noise. However, given that the measured calculation efficiency in absence of noise does not correspond to the measured calculation efficiency in high localized noise (as suggested by the current findings), both the calculation efficiency in absence of noise and the internal equivalent noise remains unknown and it is not possible to determine whether the age-related sensitivity loss in low noise was due to a lower calculation efficiency or higher internal equivalent noise.

The current study found that the most sensitive detectors underlying motion processing varied with the noise temporal window. In temporally extended noise (which includes internal noise), the most sensitive detectors were labeled for both orientation and direction, whereas in temporally localized noise, they were labeled for orientation, but not direction. In absence of noise (i.e., in internal noise), the most sensitive detectors would be labeled for both orientation and direction, which suggests, as expected, that internal noise limiting motion processing is temporally extended. Thus, to characterize motion processing in absence of noise, such as measuring internal equivalent noise and calculation efficiency, external noise should be temporally extended to avoid violating the noise-invariant processing assumption.

## Conflict of Interest Statement

The authors declare that the research was conducted in the absence of any commercial or financial relationships that could be construed as a potential conflict of interest.
